# A Bayesian Deep Neural Network for Safe Visual Servoing in Human–Robot Interaction

**DOI:** 10.3389/frobt.2021.687031

**Published:** 2021-06-17

**Authors:** Lei Shi, Cosmin Copot, Steve Vanlanduit

**Affiliations:** InViLab, Faculty of Applied Engineering, University of Antwerp, Antwerp, Belgium

**Keywords:** safety, human–robot interaction, Bayesian neural network, deep learning, image-based visual servoing

## Abstract

Safety is an important issue in human–robot interaction (HRI) applications. Various research works have focused on different levels of safety in HRI. If a human/obstacle is detected, a repulsive action can be taken to avoid the collision. Common repulsive actions include distance methods, potential field methods, and safety field methods. Approaches based on machine learning are less explored regarding the selection of the repulsive action. Few research works focus on the uncertainty of the data-based approaches and consider the efficiency of the executing task during collision avoidance. In this study, we describe a system that can avoid collision with human hands while the robot is executing an image-based visual servoing (IBVS) task. We use Monte Carlo dropout (MC dropout) to transform a deep neural network (DNN) to a Bayesian DNN, and learn the repulsive position for hand avoidance. The Bayesian DNN allows IBVS to converge faster than the opposite repulsive pose. Furthermore, it allows the robot to avoid undesired poses that the DNN cannot avoid. The experimental results show that Bayesian DNN has adequate accuracy and can generalize well on unseen data. The predictive interval coverage probability (PICP) of the predictions along *x*, *y*, and *z* directions are 0.84, 0.94, and 0.95, respectively. In the space which is unseen in the training data, the Bayesian DNN is also more robust than a DNN. We further implement the system on a UR10 robot, and test the robustness of the Bayesian DNN and the IBVS convergence speed. Results show that the Bayesian DNN can avoid the poses out of the reach range of the robot and it lets the IBVS task converge faster than the opposite repulsive pose.^1^

## 1 Introduction

With the development in human–robot interaction (HRI) and human–robot collaboration (HRC) fields, humans have more opportunities to work with robots closely. Safety is an important issue when designing HRI and/or HRC systems. It can be achieved *via* various approaches such as low-level control of robots, motion planning, and human action/motion prediction (Lasota et al., 2017). Research works have been carried out considering the safety aspect at different levels (Lasota et al., 2017; Halme et al., 2018). In a study by Fabrizio and De Luca (2016), the authors use multiple depth cameras to calculate the distance between obstacle and robot to avoid collision in real time. In a study by Polverini et al. (2017), human skeletons are tracked to avoid collisions. In a study by Wang et al. (2017), a CNN-RNN model is used to predict the human hand motion, and a critical distance is used for optimizing the trajectory to avoid collisions. In addition to the use of cameras, other sensors are also used to detect obstacles/humans, such as IMU and a laser scanner (Safeea and Neto, 2019).

Visual servoing is a closed-loop control technique that allows the robot to move to a reference pose with regard to an object. Visual servoing can be classified into image-based visual servoing (IBVS) and position-based visual servoing (PBVS) (Chaumette and Hutchinson, 2006). IBVS compares the image at the current pose and the image at the reference pose. The visual errors are iteratively reduced, and the robot pose is converged to the reference pose. It can be used in HRI applications for grasping tasks (Shi et al., 2019).

**FIGURE 1:**
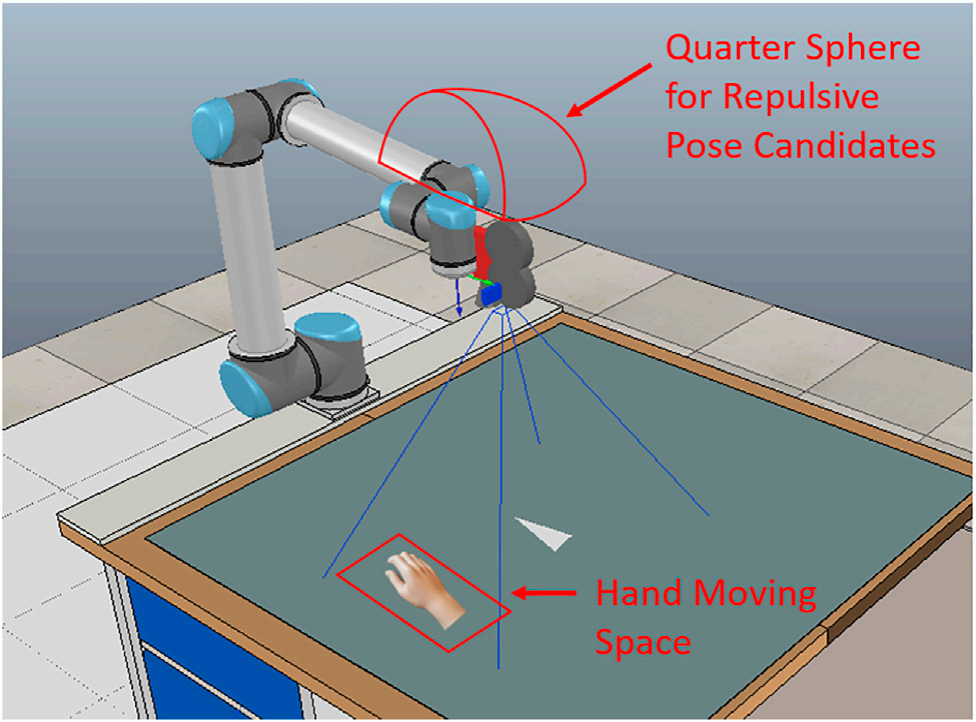
An overview of the scene setup. The camera is in eye-in-hand configuration. The object is in a triangular shape and is placed on the table. We define a space on the left side of the robot where the human hand moves. The quarter-sphere around the TCP for generating repulsive pose candidates is on the opposite direction of the hand moving space.

Considering safety when using IBVS to grasp objects, if a human hand is moving into a critical distance to the robot’s tool center point (TCP) during the process that IBVS is driving the robot to the desired pose for grasping, a repulsive action can be adopted to avoid the collision with the human (Sadeghian et al., 2015). One of the possible repulsive actions is moving the robot TCP to a repulsive pose in the opposite direction of the obstacle. As indicated in our previous work (Shi et al., 2020), moving the TCP to a repulsive pose which is around the opposite repulsive pose can still avoid the collision with the hand, and the image moment error could be smaller than the opposite pose. We use a regression ResNet (He et al., 2016; Chen et al., 2020) to predict a repulsive position. In a study by Chen et al. (2020), it is shown that using a ResNet for regression outperforms neural network (NN) and other conventional machine learning regression algorithms. As shown in Figure 1, we consider a quarter-sphere space around the robot TCP. The quarter-sphere space is on the opposite direction of the hand. From this space, we generate repulsive pose candidates and select the one with the least image error as the ground truth repulsive position. We have trained and evaluated the model using a dataset created in a simulator; the results showed that the regression ResNet can generalize well on the test set and the mean absolute errors (MAEs) in *x*, *y*, and *z* directions are 7.46, 7.61, and 7.63, respectively. It is also possible to calculate the image moment errors of the repulsive candidates online. We have shown that using the NN is faster than calculating online.

When implementing the deep neural network (DNN) model to the real system, the model uncertainty needs to be taken into consideration, especially when dealing with safety. The DNN, that is, a regression ResNet, does not provide the weight uncertainty; hence, it cannot provide prediction uncertainty. When the robot TCP moves to the space that is not included in the training data, the DNN may have undesired predictions, and the robot may have an undesired action, for example, out of the reach range. Bayesian neural networks (BNNs), on the other hand, take the uncertainties of the model weights into account during the training such that BNNs can make probabilistic inferences. Using a BNN for collision avoidance will let the system be more robust in terms of avoiding the undesired poses for the robot.

**FIGURE 2:**
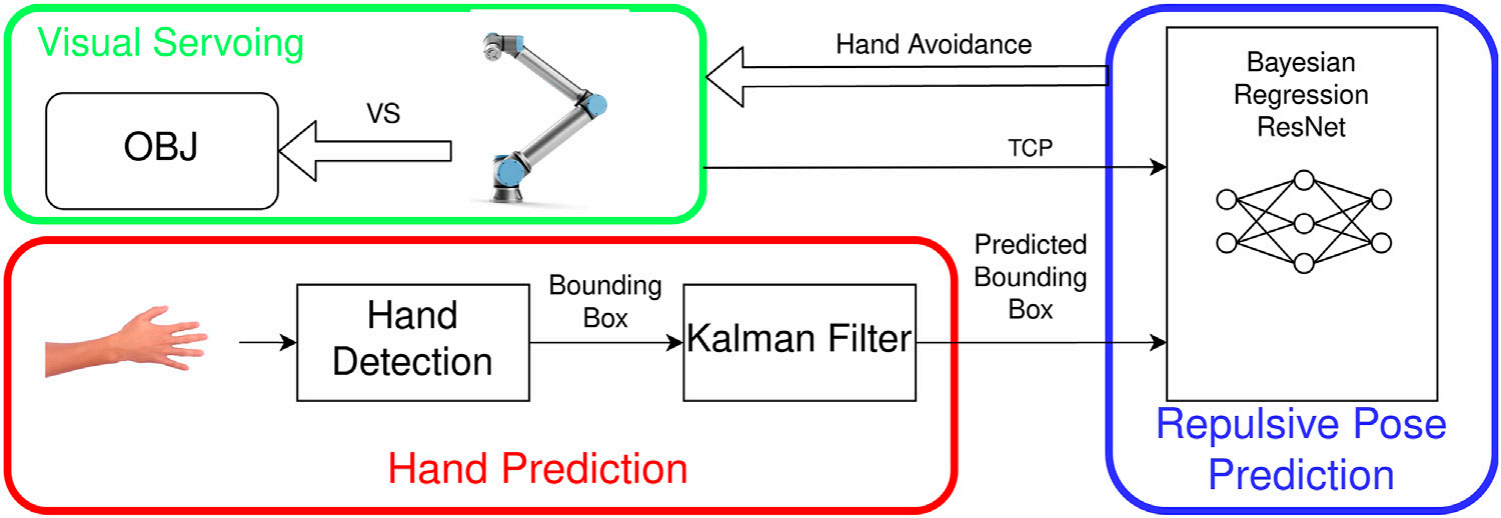
Overview diagram of the system for safe interaction with the robot.

We further develop the previous work (Shi et al., 2020). In this work, we describe a system that can avoid the collision with human hands while the robot is executing an IBVS task. The system consists of three modules, visual servoing, hand prediction, and repulsive pose prediction (Figure 2). In the visual servoing module, IBVS with image moments is used to guide the manipulator to the object of interest. The hand prediction module detects human hands and predicts the hand motion. The repulsive pose prediction module uses a Bayesian DNN based on the regression ResNet to predict the repulsive pose. We apply a Monte Carlo dropout (MC dropout) (Gal and Ghahramani, 2016) to convert the DNN into Bayesian DNN. If the predicted hand position is within a defined critical distance toward the robot TCP, the manipulator will avoid the human hand by moving the end effector to the inferred repulsive pose. We train and evaluate the Bayesian DNN, that is, a Bayesian regression ResNet, with the synthetic data created in simulation. The results show that the Bayesian regression ResNet has adequate accuracy on the test data. The predictive interval coverage probability (PICP) along *x*, *y*, and *z* axes is 0.84, 0.94, and 0.95, respectively. The Bayesian regression ResNet also has a more robust performance than the regression ResNet when the TCP is not in the range of the training data. We further implement the system with a real robot and test the inferred repulsive pose and the opposite repulsive pose. The result indicates that the IBVS can converge faster by using the inferred repulsive pose for hand avoidance.

The main contribution of our work is twofold. First, we present a system using a Bayesian regression ResNet for collision avoidance by moving the robot to a repulsive pose in IBVS; compared to the DNN, the Bayesian DNN can avoid undesired repulsive poses which will let the robot be out of its reach range. Second, we demonstrate a training method for the DNN (and Bayesian DNN) model which takes both safety and the efficiency of visual servoing tasks into consideration. The model learns a repulsive position at which the image has the least visual error regarding the desired pose. The repulsive pose based on the Bayesian DNN ensures safety by moving in the opposite direction of the obstacle, and it lets the IVBS converge faster than the direct opposite repulsive pose. The rest of the article is organized as follows: in *Related Work*, we review the related work; in *System Overview* and *Implementation*, we explain the details of the system and its modules as well as the implementation; in *Results*, we show the evaluation results of the Bayesian regression ResNet and the results of the system, and we discuss the results in *Discussion*. The conclusion and future work are shown in *Conclusion and Future Work*.

## 2 Related Work

In a study by Khatib (1986), a potential field approach is used for collision avoidance. The potential field calculation is based on the distance, and the potential field is used to transform the control problem into a velocity-based servo control. In a study by Flacco et al. (2012), a distance-based repulsive vector is used for collision avoidance in 3-dimensional space in various scenarios. The depth is obtained by a RGB-D camera. In a study by Fabrizio and De Luca (2016), multiple depth cameras are used to estimate the distance between the obstacles and the points of interest on the robotic link. The obstacle avoidance is achieved by considering the distances and orientations between the obstacles and the points on the robotic link. In a study by Polverini et al. (2017), the authors use a kinetostatic safety field to control the robot for collision avoidance. The kinetostatic safety field considers both the relative position and the relative velocity between an obstacle and points on a robot. In a study by Sadeghian et al. (2015), the authors use a distance-based repulsive action during image moment–based IBVS and keep the object at the center of the image.

With rapid developments in artificial intelligence, it draws attention to the robotics research works too. Several works by Heo et al. (2019), Sharkawy et al. (2020), Cioffi et al. (2020), and Anvaripour and Saif (2019) deployed artificial intelligence techniques in the safety issues in HRI. In a study by Sharkawy et al. (2020), a multilayer feedforward NN is used to detect a collision and identify the collided robotic link. In a study by Cioffi et al. (2020), a support vector machine (SVM) classifier is used to classify if contact to the robot is intentional or is a collision. In a study by Heo et al. (2019), a DNN model, CollisionNet, is proposed to detect collision. The model takes joint’s information and acts as a binary classifier to predict if a collision happens or not. The authors in Anvaripour and Saif (2019) use CollisionNet in combination with force myography sensors attached to the human arm to classify if a collision is intended or not.

Different approaches have been proposed, allowing the NN to make Bayesian inference, such as Bayes by Backprop (Blundell et al., 2015), Bayesian hypernetworks (Krueger et al., 2017), multiplicative normalizing flows (Louizos and Welling, 2017), Bayes by Hypernet (Pawlowski et al., 2017), and dropout as a Bayesian optimization (MC Dropout) (Gal and Ghahramani, 2016). Bayes by Backprop learns the distribution of weights in a NN. The weights are regularized by the variational free energy. Bayesian hypernetwork consists of two parts, a hypernetwork and a primary network, that is, the NN of interest. The hypernetwork learns the parameters of the primary network, and they are trained together by backpropagation. Dropout as a Bayesian optimization uses dropout to approximate the Bayesian inference for a NN. From the perspective of integrating a Bayesian DNN into real robotics application, dropout as a Bayesian optimization has the advantage of easy implementation upon an existing network by simply adding dropout layers to the weight layers. MC dropout has been widely adopted in various research fields. Applications include camera pose estimation (Kendall and Cipolla, 2016), depth estimation (Poggi et al., 2020), pedestrian localization (Bertoni et al., 2019), semantic segmentation (Mukhoti and Gal, 2018), and electrocardiogram signal detection (Elola et al., 2019).

A more similar work to ours is the work of Kahn et al. (2017); the authors applied bootstrapping and dropout on an NN to estimate the uncertainty of collision in unmanned aerial vehicle (UAV) and autonomous car applications. However, our work has the following differences with that of Kahn et al. (2017). First, the NN they used takes the current state, observation, and control as inputs, and provides the probability of collision as the output. In our work, we modified the ResNet for a regression task, which takes the hand position and TCP position as inputs, and provides the repulsive position as the output. Second, the Bayesian NN in the work of Kahn et al. (2017) is further used for reinforcement learning for collision avoidance. Our Bayesian DNN is directly used for collision avoidance with shorter convergence iteration for IBVS. In a study by Michelmore et al. (2020), the authors also used MC dropout on an end-to-end DNN to control the vehicle in autonomous driving. Unlike in the work of Kahn et al. (2017), the DNN acts as an end-to-end controller which takes the images as inputs and provides the steering angles for the car as the output. The MC dropout technique is only applied on the last three fully connected (FC) layers. The MC drop is also used in a study by Cui et al. (2019) in an end-to-end manner for training.

## 3 System Overview

Figure 2 gives an overview of the system for safe interaction with the robot. In the visual servoing module, IBVS with image moments is used to locate the object. The camera is configured as the eye-in-hand system. The hand prediction module detects the human hand and predicts its motion. The module determines if the hand is within the critical distance. The repulsive pose prediction takes two inputs, the TCP of the end effector and the predicted position of the hand. If the hand is in the range of the critical distance, a repulsive pose will be predicted by the Bayesian regression ResNet. The robot will move to the repulsive pose to avoid the potential collision with the hand.

### 3.1 IBVS With Image Moments

One of the most common controllers used in IBVS applications is the proportional control law (Chaumette et al., 2016; Copot et al., 2019), which is defined as follows:

$$v_{c}=-\lambda L_{f}^{+}e,$$ (1)

where $L_{f}^{+}$ is the Moore–Penrose pseudoinverse of the interaction matrix $L_{f}$ attached to the visual features **f**. In this study, we make use of image moments as visual features. A set of image moments $f=\left[x_{n},y_{n},a_{n},\gamma ,\delta ,\alpha \right]$ is used to design the control law. In order to design this control law, an exponential decrease of the error $\dot{e}=-\lambda e=-\lambda \left(f-f^{\text{*}}\right)$ has been considered. The interaction matrix $L_{f}$ for a set of image moments **f** is given by Tahri and Chaumette (2005):

$$L_{f}=\left[\begin{array}{cccccc} -1 & 0 & 0 & a_{n}e_{11} & -a_{n}\left(1+e_{12}\right) & y_{n}\\ 0 & -1 & 0 & a_{n}\left(1+e_{21}\right) & -a_{n}e_{11} & -x_{n}\\ 0 & 0 & -1 & -e_{31} & e_{32} & 0\\ 0 & 0 & 0 & \gamma_{\omega x} & \gamma_{\omega y} & 0\\ 0 & 0 & 0 & \delta_{\omega x} & \delta_{\omega y} & 0\\ 0 & 0 & 0 & \alpha_{\omega x} & \alpha_{\omega y} & -1 \end{array}\right].$$ (2)

The parameters from the interaction matrix are calculated as follows:

$$x_{n}=a_{n}x_{g},\;y_{n}=a_{n}y_{g},\;a_{n}=Z*\sqrt{\frac{a*}{a}},$$ (3)

$$\gamma =\frac{I_{n1}}{I_{n3}},\;\delta =\frac{I_{n2}}{I_{n3}},\;\alpha =\frac{1}{2}\text{arctan}\left(\frac{2\mu_{11}}{\mu_{20}-\mu_{02}}\right),$$ (4)

where

$$\begin{array}{l} a=\mu_{20}+\mu_{02},\\ I_{n1}={\left(\mu_{50}+2\mu_{32}+\mu_{14}\right)}^{2}+{\left(\mu_{05}+2\mu_{23}+\mu_{41}\right)}^{2},\\ I_{n2}={\left(\mu_{50}-2\mu_{32}-3\mu_{14}\right)}^{2}+{\left(\mu_{05}-2\mu_{23}-3\mu_{41}\right)}^{2},\\ I_{n3}={\left(\mu_{50}-10\mu_{32}+5\mu_{14}\right)}^{2}+{\left(\mu_{05}-10\mu_{23}+5\mu_{41}\right)}^{2}, \end{array}$$ (5)

and $Z^{\text{*}}$ is the desired depth between the visual sensor and the desired configuration.

The centered moments $\mu_{ij}$ are computed by

$$\mu_{ij}=\overset{n}{\underset{k=1}{\sum }}{\left(x_{k}-x_{g}\right)}^{i}{\left(y_{k}-y_{g}\right)}^{j},$$ (6)

with $x_{g}=\frac{m_{10}}{m_{00}}$, $y_{g}=\frac{m_{01}}{m_{00}}$, and $m_{ij}=\overset{n}{\underset{k=1}{\sum }}x_{k}^{i}y_{k}^{j}$.

### 3.2 Hand Detection and Prediction

To detect the human hands, we use a deep learning–based object detector, that is, YOLO (Redmon et al., 2016). The object detector is trained on the COCO dataset (Lin et al., 2014). The detected object is formed of $O=\left[x_{b},y_{b},h_{b},w_{b}\right]$, where $x_{b}$ and $y_{b}$ are the centers of the bounding box, and $w_{b}$ and $h_{b}$ are the width and height of the bounding box, respectively. To predict the bounding box of the hand, a Kalman filter is implemented (Bewley et al., 2016). The state vector of the Kalman filter is given below:

$$x_{s}=\left[x_{b},y_{b},z_{b},a_{b},r_{b},{\dot{x}}_{b},{\dot{y}}_{b},{\dot{z}}_{b},{\dot{a}}_{b}\right],$$ (7)

where $z_{b}$ is the depth at the center of the bounding box, $a_{b}$ is the area of the bounding box, and $r_{b}$ is the aspect ratio. We take the center of the predicted bounding box ${\hat{t}}_{b}=\left[{\hat{x}}_{b},{\hat{y}}_{b},{\hat{z}}_{b}\right]$ as the predicted position of the hand, where ${\hat{x}}_{b}$ and ${\hat{y}}_{b}$ are the coordinates in the camera coordinate system. ${\hat{t}}_{b}$ is converted to the TCP coordinate system ${\hat{t}}_{h}$ by using the pinhole camera model.

### 3.3 Repulsive Pose Prediction

We use a regression ResNet model (Chen et al., 2020) to predict the repulsive pose. The results in the study by Shi et al. (2020) have shown that the MAEs of the model on the test set are less than 8. For allowing the NN to make Bayesian inference, we apply the MC dropout technique (Gal and Ghahramani, 2016) to the regression ResNet.

#### 3.3.1 ResNet for Regression

**FIGURE 3:**
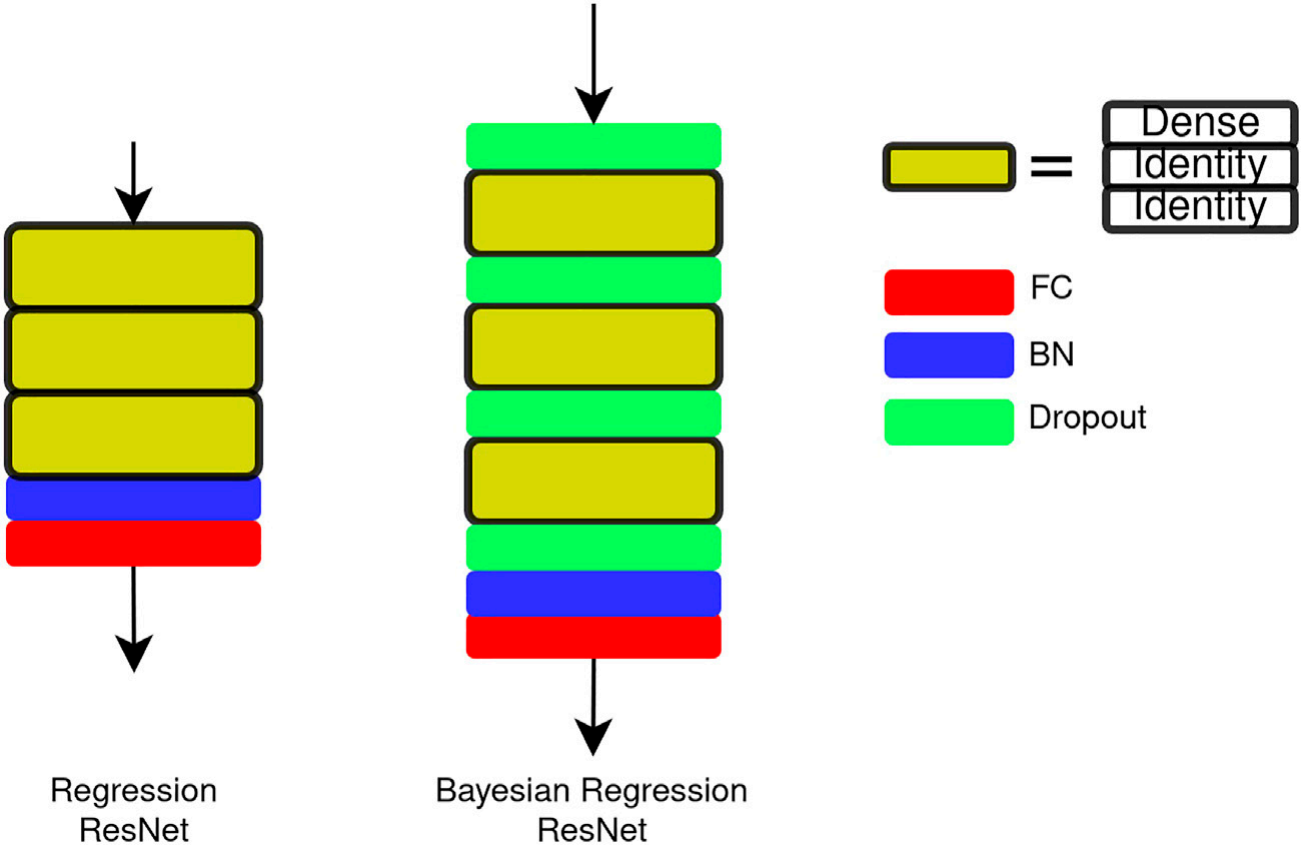
Architecture of the regression ResNet and the Bayesian regression ResNet. The regression ResNet consists of three residual blocks (yellow) and a BN + FC as readout. The residual block consists of one dense block and two identity blocks. The Bayesian regression ResNet is built upon the regression ResNet, and dropout layers are inserted between residual blocks.

**FIGURE 4:**
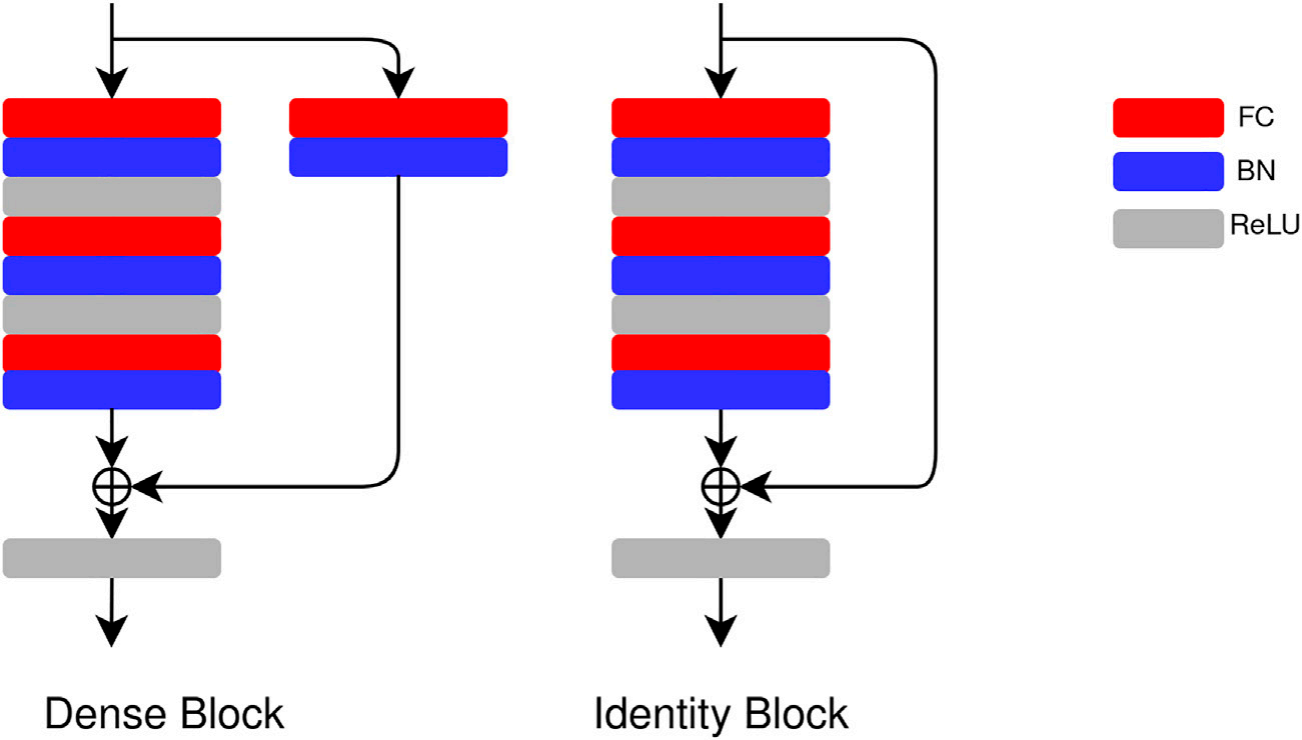
Architecture of the dense block and the identity block in the regression ResNet and the Bayesian regression ResNet. In a dense block, there is FC + BN in the residual connection, and there is no FC + BN in the residual connection in an identity block.

We use a ResNet-based network for the regression task (Chen et al., 2020). The architecture of the network is shown in Figure 3. Three residual blocks are connected in series, and the output of the stacked blocks is passed to a batch normalization (BN) (Ioffe and Szegedy, 2015) layer and an FC layer. In one residual block, two types of blocks, that is, dense block and identity block, are used. The details of the blocks are shown in Figure 4. The main difference between the ResNet and the regression ResNet is that the convolutional layers and pooling layers in the ResNet are replaced by FC layers. The FC layers are followed by the BN layers and rectified linear units (ReLUs) (Nair and Hinton, 2010).

The input of the model is the predicted hand position ${\hat{t}}_{h}$ and the current TCP position $t_{tcp}$ of the robot. We expand the input with one additional bias element, so the dimension of the input is 7. The output of the regression ResNet is the position of the repulsive pose, and its dimension is 3. The orientation of the repulsive pose is the same as the current TCP orientation.

#### 3.3.2 MC Dropout

For an NN with weight W, bias b, and loss function E(⋅,⋅), the cost function ℒ is as follows:

$$ℒ=\frac{1}{N}\overset{N}{\underset{i=1}{\sum }}E\left(y_{i},{\hat{y}}_{i}\right)+\lambda_{d}\overset{L}{\underset{i=1}{\sum }}\left({\left\|W_{i}\right\|}_{2}^{2}+{\left\|b_{i}\right\|}_{2}^{2}\right),$$ (8)

where y and $\hat{y}$ are the ground truth and output of an NN, respectively, and *N* is the length of the training data. The second term is a $L_{2}$ regularization term with decay factor $\lambda_{d}$. When the dropout operation is applied, some weights in the network are removed following a Bernoulli distribution. As the title suggests (Gal and Ghahramani, 2016), the authors proved that using dropout in an NN is equivalent to approximate Bayesian inference in deep Gaussian processes. It then gives the uncertainty in an NN. For approximating the Gaussian process model, variational inference is used, and Monte Carlo integration is used for minimizing Kullback–Leibler (KL) divergence. The cost function is scaled to

$${ℒ}_{GP-MC}\propto \frac{1}{2N}\overset{N}{\underset{i=1}{\sum }}E\left(y_{i},{\hat{y}}_{i}\right)+\lambda_{d}\overset{L}{\underset{i=1}{\sum }}\left[\frac{\left(1-p_{i}\right)l^{2}}{2\tau N}{\left\|W_{i}\right\|}_{2}^{2}+\frac{l^{2}}{2\tau N}{\left\|b_{i}\right\|}_{2}^{2}\right],$$ (9)

where $p_{i}$ is the dropout rate in the *i*th layer, *τ* is the model precision, and *l* is the prior length scale. The predictive mean and predictive variance can be obtained by iterating the NN forward process *T* times, as given below:

$$E\left(y^{*}\right)\approx \frac{1}{T}\overset{T}{\underset{i=1}{\sum }}{\hat{y}}^{\text{*}}\left(x^{\text{*}},W_{1}^{i},\ldots ,W_{L}^{i}\right),$$ (10)

$$Var\left[\left(y^{\text{*}T}\right)\left(y\right)\right]\approx \frac{1}{T}\overset{T}{\underset{i=1}{\sum }}{\hat{y}}^{\text{*}}{\left(x^{\text{*}},W_{1}^{i},\ldots ,W_{L}^{i}\right)}^{T}{\hat{y}}^{\text{*}}\left(x^{\text{*}},W_{1}^{i},\ldots ,W_{L}^{i}\right),$$ (11)

where E is the predictive mean and Var is the predictive variance. The weight decay $\lambda_{d}$ is calculated by the following equation:

$$\lambda_{d}=\frac{pl^{2}}{2N\tau }.$$ (12)

However, associating each weight layer with a dropout layer will let the regularization be too strong during the training for the network (Alex Kendall and Cipolla, 2017), which will result in a long training process. Hence, we adapt dropout layers to the regression ResNet as in Figure 3. A dropout layer is inserted between each stacked residual block in the network. We demonstrate that associating each weight layer with a dropout layer is not necessary for the regression ResNet in *Dropout Placement*.

## 4 Implementation

We use the same method as in the study by Shi et al. (2020) to create the training data. The training data are generated in a simulator instead of collecting the data from the real robot. The advantage is that the time used will be significantly shorter (Bateux et al., 2018). We limit the hand position and TCP position within certain ranges. The repulsive pose candidates are limited to a quarter-sphere space which is in the opposite direction of the hand and the TCP.

### 4.1 Training

The training data for the DNN and Bayesian DNN are created in a simulator. The simulation environment is generated in CoppeliaSim, and MATLAB is used as the interface to the simulation environment. Figure 1 shows the overview of the simulation environment. We use a triangular-shaped object. The object is placed on the table. For the IBVS, a desired image is required so that the robot can move to the desired pose for further actions. We first take a desired image at the desired pose. Then a set of 10,000 TCP poses around the desired pose is considered for the visual servoing task, and for each of the TCP pose, 100 repulsive pose candidates are generated. Each TCP pose candidate has a related image and a related set of visual features. From the 10,000 TCP poses, we select 1,000 poses and generate real data samples in the real robot setup. We validate the synthetic data with the real data to eliminate the errors between the real setup and the simulation setup.

**FIGURE 5:**
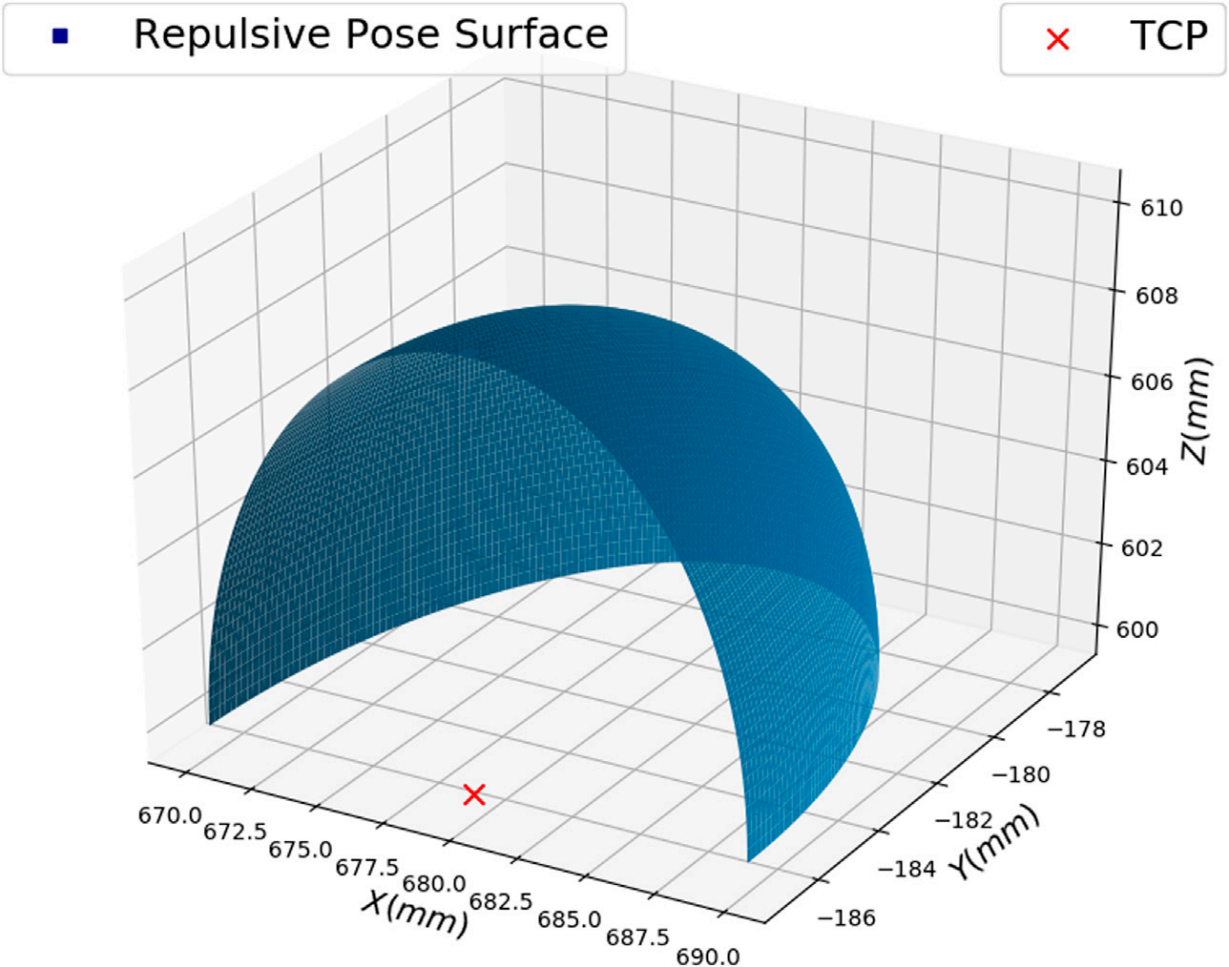
An example of repulsive pose candidates for a defined TCP pose.

The repulsive pose candidates are in the opposite direction of the hand position and are distributed on a quarter of a sphere with respect to the TCP pose. We did not consider the whole sphere since our motivation is to find a repulsive pose around the direct opposite repulsive pose that can avoid the collision with the hand. If the whole sphere is considered, we may select a repulsive candidate which will lead the robot to move toward the hand. Using a quarter of a sphere will lead the robot to move away from the hand. The candidates’ distribution follows a Gaussian distribution. Figure 5 shows an example. We process the images taken from the repulsive pose candidate to obtain the image visual error. We first apply thresholding to get the binary image. Then the image moments are extracted based on Eq. 6. Next, we calculate the image moment errors between each of the 100 repulsive pose candidates and the desired pose. The selected repulsive pose candidate is the one that has the least $L_{2}$ image moment error. The position of the hand can deviate with ± 300 mm from its initial position (840,−350,5) with respect to the robot base in mm. In total, 500 hand positions are generated. For each hand position, it is combined with the 10,000 TCP poses. The training data thus will have 5,000,000 samples in total.

The selected repulsive pose candidates are processed to get the ground truth for the training. The final ground truth is calculated as follows:

$$gt=0.05\times \left(t_{tcp}-t_{h}\right)+t_{opt},$$ (13)

where $t_{tcp}$ is the position of robot TCP and $t_{opt}$ is the position of the selected repulsive pose candidate.

The training data are divided into a training set, a validation set, and a test set, and the split ratio is 0.8, 0.1, and 0.1, respectively. The sizes of the training set, the validation set, and the test set are 4,000,000, 1,000,000, and 1,000,000, respectively. The Bayesian DNN is trained with 200 epochs. The batch size is 2000. $L_{1}$ loss function and Adam optimizer are used for the training. The learning rate is 0.001. The weight decay of the optimizer $\lambda_{d}$ is calculated by Eq. 12. The model precision *τ* is 1e−6, the prior length scale *l* is 0.01, and the dropout rate *p* is 0.1 for all dropout layers. The hidden units of FC layers are 32. After each epoch, the model is validated, and the final model is the one with the least validation loss.

### 4.2 Implementation Detail

To implement the total system, we use Universal Robots UR10 robot and ROS for controlling the UR10. The RealSense D435 camera is used to obtain RGB-D images. The YOLO object detector is also implemented in ROS (Bjelonic, 2018). VISP (Marchand et al., 2005) is used for IBVS with image moments. The model is trained with PyTorch. The trained model is then converted to a C++ model so that it can be integrated into the robotic system.

## 5 Results

We demonstrate the evaluation of the Bayesian regression ResNet in this section. We perform experiments on test datasets generated in the simulator and experiments on the real robot. In the experiments on test datasets, we test model parameters and the model architecture. We further compare the performances of Bayesian DNN and DNN on the test datasets whose space is unseen. In the experiments on the real robot, we first see the performances of Bayesian DNN and DNN on the real robot. Next, we compare the IBVS convergence speed by applying the Bayesian DNN and the opposite repulsive pose. The results are organized as follows. First, the Bayesian regression ResNet is evaluated on the test dataset to see how differently *T* (*Inference Iterations*) and dropout placement (*Dropout Placement*) affect the model. Next, we see how the Bayesian regression ResNet will perform in the unseen space in *Data From Unseen Space*. We generate new test datasets whose data are not in the range of the original test dataset, and compare the Bayesian regression ResNet with the regression ResNet. Then we conduct experiments with the real robot to see the performance. Last, we evaluate the convergence speed in *Comparison With Opposite Repulsive Pose*.

### 5.1 Inference Iterations

In this subsection, we show the effect of different inference iterations *T*. As indicated in Eq. 10, forwarding the input to the Bayesian DNN *T* times is necessary to make Bayesian inference. The evaluation is performed on the test set (*Implementation*). The PICP and the root mean square error (RMSE) are used to evaluate the model with different inference iterations *T*. The PICP is calculated as follows:

$$PICP=\frac{Number\;of\;predictions\;in\;prediction\;intervals}{Total\;number\;of\;predictions}.$$ (14)

The detailed calculation is described in the study by Pearce et al. (2018).

**FIGURE 6:**
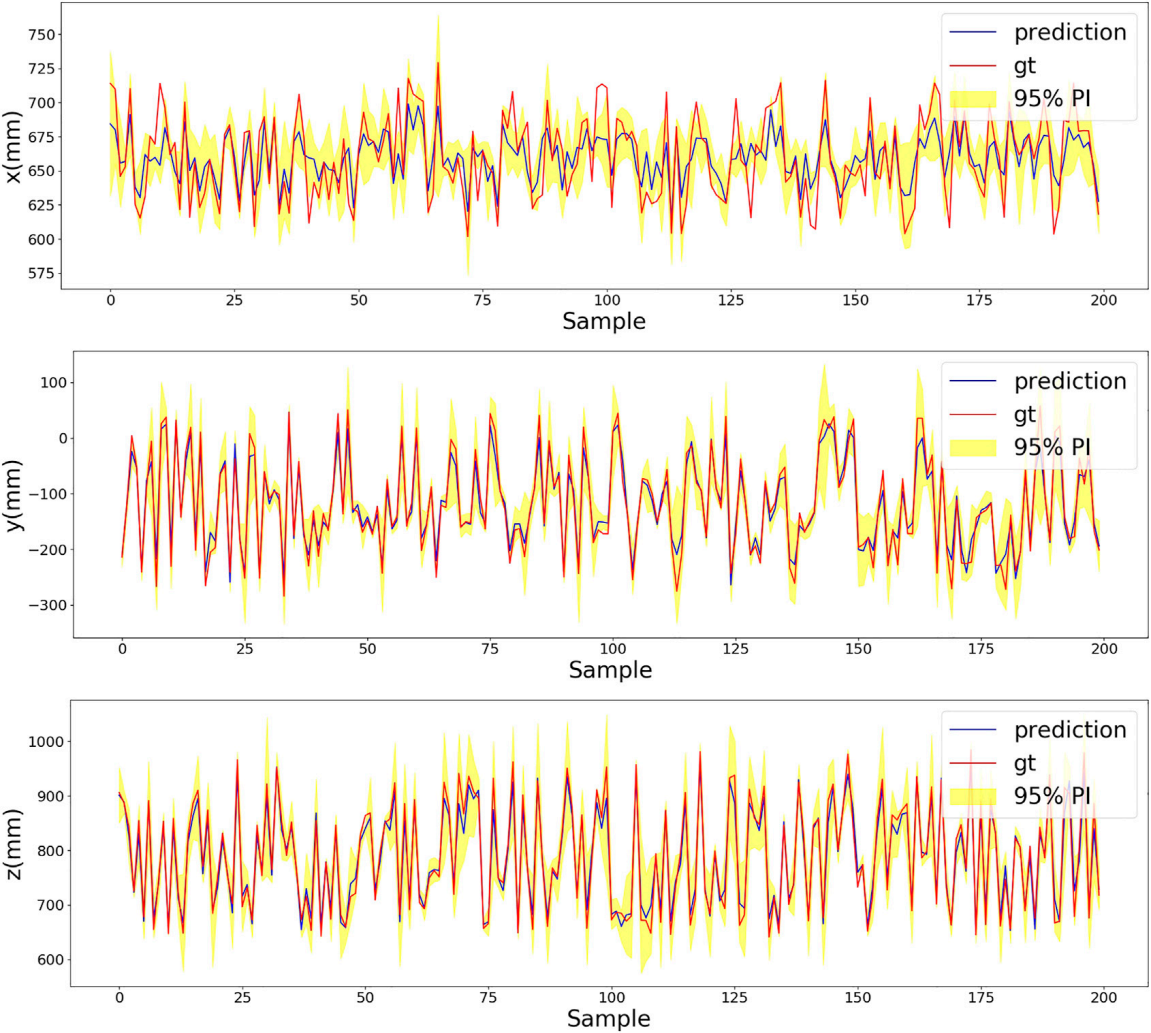
Plots of x, y, and z of the first 200 samples in the test data. The blue solid line represents the mean prediction of the Bayesian regression ResNet and the red dotted line is the ground truth. The yellow region is 95% of the prediction interval.

Table 1 shows the PICP for the prediction in the single-axis direction and the RMSE. With the increased *T*, the PICP and RMSE get better. However, after the inference iteration is higher than 10, the improvements on the PICP and RMSE get smaller. In the meantime, a higher number of iterations mean longer inference time, which needs to be considered in real-time applications. Figure 6 provides a visualization example for the results in Table 1. The plots display the first 200 samples in the test data. The inference running iteration T=10. For most of the mean predictions, the ground truth falls in the prediction interval. Selecting T=10 is adequate for making Bayesian inference.

**TABLE 1:** RMSE and PICP of different inference iterations *T*.

	T = 5	T = 10	T = 15	T = 20	T = 25
PICP (x)	0.8	0.84	0.85	0.85	0.85
PICP (y)	0.9	0.94	0.96	0.96	0.97
PICP (z)	0.91	0.95	0.97	0.98	0.98
RMSE	21.93	20.41	19.87	19.6	19.2

### 5.2 Dropout Placement

We see the performance of two different dropout placements, that is, DO-Inter and DO-Full in the network architecture. DO-Inter refers to the arrangement that dropout layers are placed between residual blocks, as shown in Figure 3. DO-Full associates a dropout layer with each FC layer. These two dropout arrangements are evaluated on the test set (Section 4). The inference iteration is T=10 for DO-Inter and DO-Full. Table 2 summarizes the results. DO-Full is trained with 1,200 epochs, and DO-Inter is trained with 200 epochs. DO-Full has a longer training process, and the performance on the test set is lower than that of DO-Inter. For a regression ResNet architecture, it is not necessary to associate a dropout layer with each FC layer in order to transform the DNN into the Bayesian DNN.

**TABLE 2:** RMSE and PICP of different placements of dropout layers.

	PICP (x)	PICP (y)	PICP (z)	RMSE	Epoch
DO-inter	0.84	0.94	0.95	20.41	200
DO-full	0.71	0.94	0.94	22.49	1,200

As indicated in Table 1, 2, the PICPs in *x* direction are lower than those in *y* and *z* directions. In the training data, the range in *x* direction is smaller than that in *y* and *z* directions. This causes the lower PICP in *x* direction. When we create the training data, the object needs to be in the image. If the range in *x* direction increases, the object will not appear in the image.

### 5.3 Data From Unseen Space

The data used to train the model are limited to certain spaces. Additionally, we form three new test sets to see how the regression ResNet and the Bayesian regression ResNet model will perform on the data that are out of the range of the data we used for training, validation, and test. In the first new test set, the hand space is out of range and the TCP space is within the same range. In the second new test set, the hand space is the same and the TCP space is out of range; and in the third one, both the hand space and the TCP space are out of range. The data for training the regression ResNet are the same as the data for training the Bayesian regression ResNet. The training details are the same as in the study by Shi et al. (2020). The inference iteration *T* for the Bayesian regression Resnet is 10.

Table 3 shows the results of the test on the new data whose space is out of the range from the dataset used for training. When the hand space is out of range, the PICP and RMSE of the Bayesian regression ResNet and the regression ResNet are comparable with the ones that are tested in the seen space. When the test of the TCP is out of range as well as both the hand and TCP are out of range, the PICPs and RMSEs of both models are worse than the situation when only the hand space is out of range. The RMSE of the regression ResNet is smaller than that of the Bayesian regression ResNet when the TCP is in the seen space. On the other hand, when TCPs are out of the range of the simulation-generated TCPs used for the training, validation, and test, the RMSEs of the regression ResNet are larger than those of the regression ResNet. The Bayesian regression ResNet has a more robust performance when the robot TCP is out of range.

**TABLE 3:** RMSE and PICP of the test in the unseen space. DNN is the regression ResNet and the Bayesian DNN is the Bayesian regression ResNet.

		Hand	TCP	Hand and TCP
Bayesian DNN	PICP (x)	0.84	0.69	0.69
	PICP (y)	0.94	0.68	0.68
	PICP (z)	0.95	0.76	0.76
	RMSE	20.58	22.53	22.73
DNN	RMSE	11.43	28.41	28.59

Next, we compare the regression ResNet and the Bayesian DNN on the real robot. In the experiment, we need the hand positions in two trials and the triggering time of hand avoidance to be exactly the same in order to evaluate the regression ResNet and the Bayesian DNN quantitatively. But it is not feasible in the real experimental setting. Hence, we conduct the experiment with the following steps: first, we use a fixed hand position for all the trials in the experiment; second, we trigger the hand avoidance at the same iteration for both networks. We test the two networks in 50 TCP positions which are not in the range of the dataset created in the simulator. Table 4 shows the result on the real robot. For the regression ResNet, 22 out of 50 of the repulsive poses based on regression ResNet predictions are out of the reach range of the robot. All repulsive poses based on Bayesian DNN predictions are within the reach range of the robot.

**TABLE 4:** Performances of the regression ResNet and the Bayesian DNN on the real robot.

	Trials out of robot reach range	Total trials
Regression ResNet	22	50
Bayesian DNN	0	50

### 5.4 Comparison With the Opposite Repulsive Pose

We evaluate the repulsive pose based on Bayesian DNN prediction with the opposite repulsive pose by comparing the convergence iteration of IBVS. The gain *λ* of IBVS (Eq. 1) determines how fast the robot will react to the visual error. At the beginning of the IBVS task, the visual error is large, and large *λ* enables the robot to move faster toward the desired pose. When the robot is close to the desired pose, the large *λ* will let the robot oscillate around the desired pose. Thus, we set λ=0.4 when the IBVS task is started, and we calculate the sum squared error of image moments **f**, as follows:

$$sse={\left\|f\right\|}^{2}.$$ (15)

**FIGURE 7:**
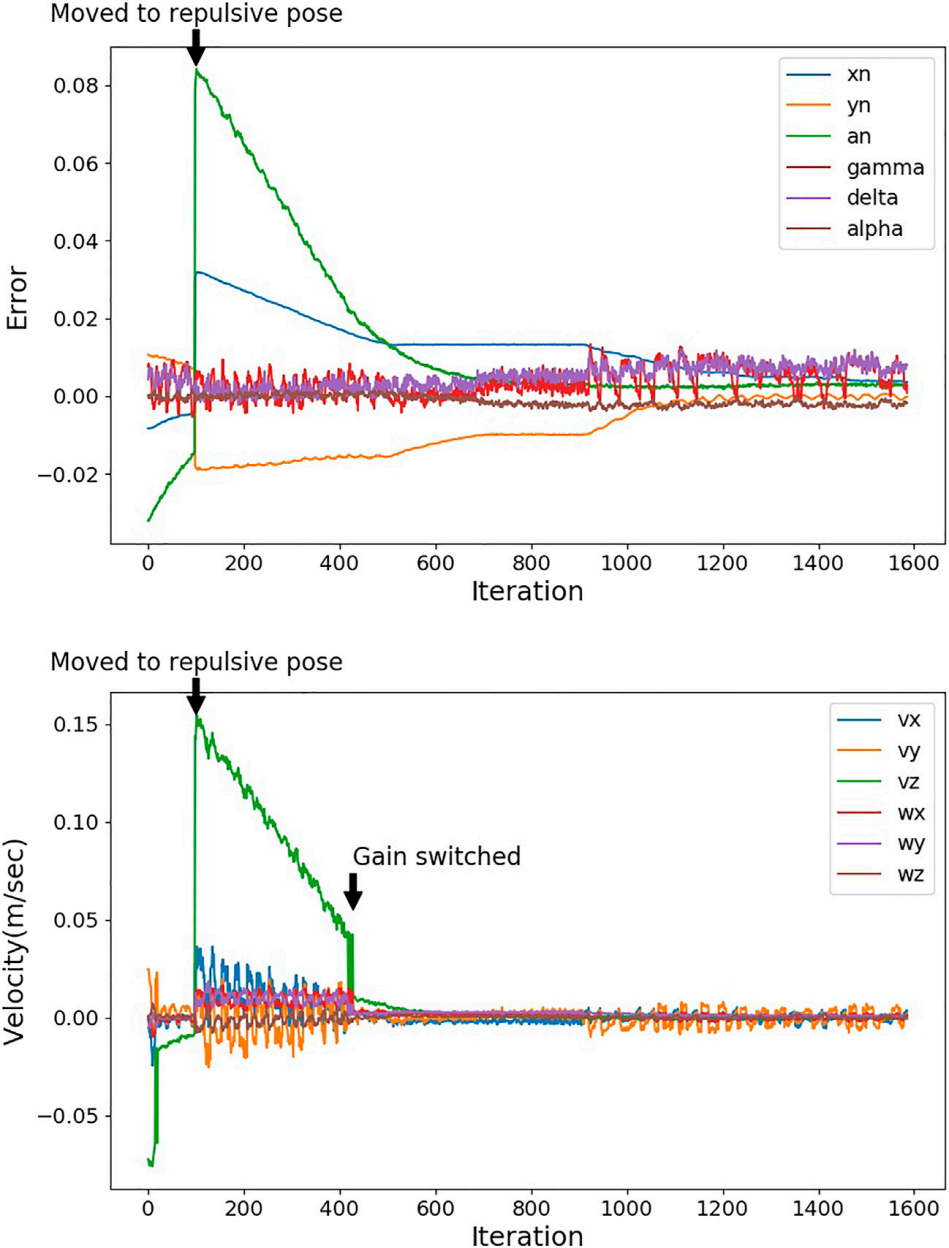
Image moments **(top)** and output velocities **(bottom)** of IBVS. At the 100th iteration, the hand position is predicted within the critical distance to the TCP, and a repulsive pose is applied to the robot. At the 428th iteration, the gain is switched from 0.4 to 0.1.

When sse is smaller than 0.005, we set λ=0.1. Figure 7 shows an example of the trail on the real robot. The image moments and output velocity during the IBVS task are indicated in the figure. From iteration 0 to 100, the IBVS task is in execution; this means the robot is moving to the desired pose for further action. At the 100th iteration, a hand is detected within the critical range, and a repulsive pose is generated. The robot pauses the IBVS and moves to the repulsive pose to avoid the collision with the hand. When the robot has moved to the repulsive pose, the IBVS is continued. At the 428th iteration, the sse is smaller than 0.005, and *λ* is switched to 0.1.

**FIGURE 8:**
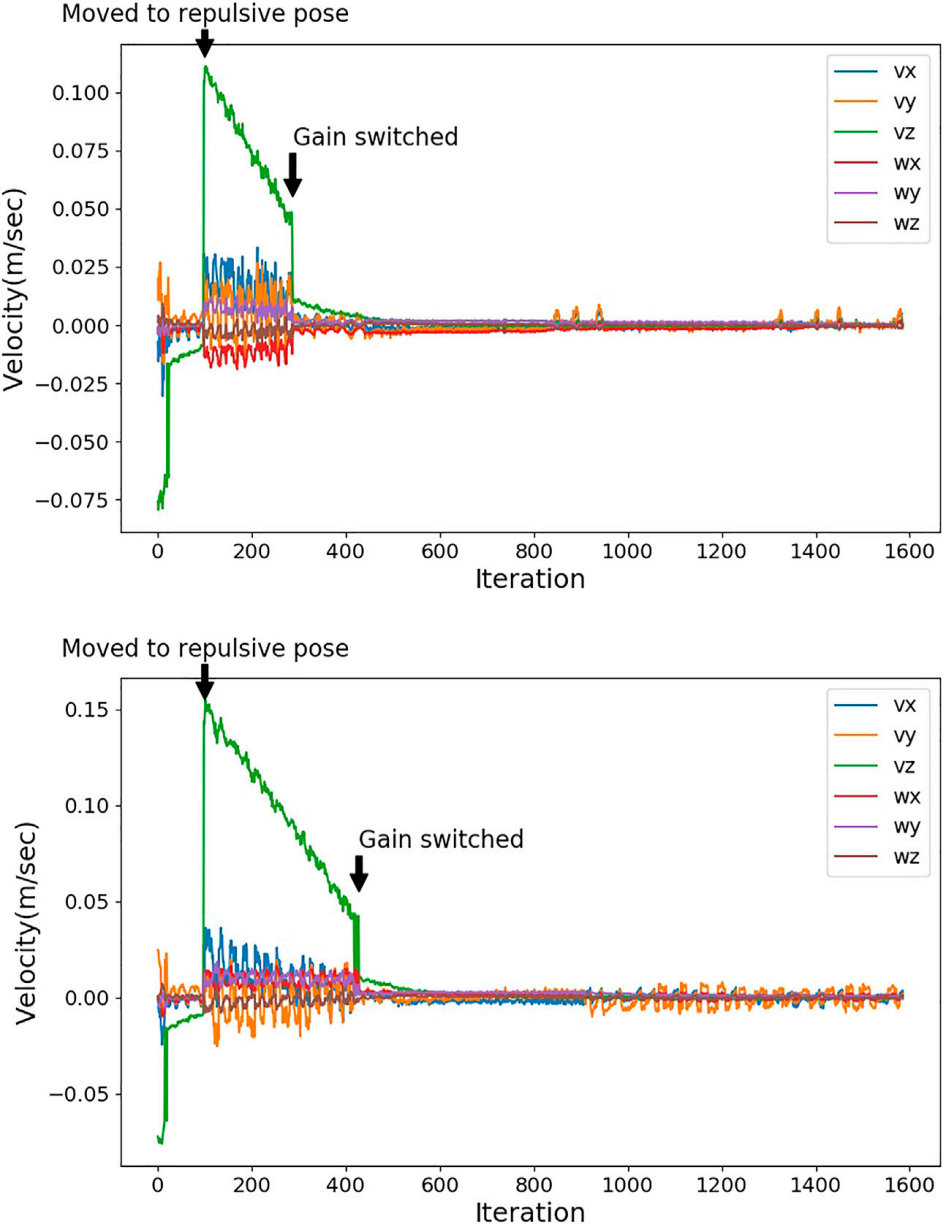
The top plot is the output velocities of the repulsive pose based on Bayesian DNN prediction. The bottom plot is the output velocities of the opposite repulsive pose. The repulsive pose based on Bayesian DNN prediction and the opposite repulsive pose are generated at the same robot TCP, and it is moved to the repulsive pose at 100th iteration with a constant hand position. Gain is switched to 0.1 when sse is smaller than 0.005.

As described in *Data From Unseen Space*, it is difficult to perform a new trial so that the hand is detected in the exact position and at the exact time as in the previous trial. We adopt similar settings to compare the opposite repulsive pose with Bayesian DNN prediction–based repulsive pose. A constant hand position is used for both repulsive poses. In total, 20 TCP poses are tested. The TCP poses are not in the space for training the networks. For each of the 20 TCP poses, an opposite repulsive pose and a Bayesian DNN prediction–based repulsive pose are generated. Figure 8 shows an example. The repulsive pose will be calculated at the same robot TCP with constant hand position and the robot moves to the repulsive pose, then the IBVS task continues. When sse is smaller than 0.005, the gain is switched to 0.1. We evaluate the convergence speed by comparing the iterations from the robot that has moved to the repulsive pose to the gain that has switched. Table 5 shows the results of convergence speed. The convergence speed for Bayesian DNN is significantly faster than that of the opposite repulsive pose. When the hand is within the critical distance towards the robot TCP, Bayesian DNN can let the robot move to the final desired pose faster than moving to the opposite repulsive pose.

**TABLE 5:** Average convergence iterations from repulsive pose to gain switching for Bayesian DNN and opposite repulsive pose.

	Average convergence iteration	Total tested TCPs
Bayesian DNN	201.3	20
Opposite	869.28	20

## 6 Discussion

In this work, we propose a system for safety in HRI applications. When a robot is executing an IBVS task and if a human hand is moving close to the robot TCP, a repulsive pose is predicted, and the robot moves to the repulsive pose to avoid hand collision. For hand prediction, we use the YOLO object detector to detect the hand and Kalman filter to predict the motion of the detected bounding box. We did not implement state-of-the-art approaches for human motion prediction since our main focus is on predicting the repulsive position by Bayesian DNN. It does not affect our contribution to the use of Bayesian DNN.

The Bayesian regression ResNet model is used to learn a repulsive position to avoid hand collision. We generate training data in a simulator. The repulsive position should let IBVS converge faster than the opposite repulsive pose and ensure the hand avoidance at the same time. To achieve this, we generate repulsive pose candidates in a quarter-sphere range around the robot TCP. The quarter-sphere range is in the opposite direction of the hand position. From the repulsive pose candidates, we select the one with the least image moment errors regarding the IBVS desired pose. The Bayesian DNN model is transformed from the regression ResNet by adding dropout layers (Gal and Ghahramani, 2016). In the original study, a dropout layer is placed before each layer that has weights in an NN. For DNNs with various network architectures, this will lead to longer training epochs due to the strong regularization. In a study by Alex Kendall and Cipolla (2017), the authors have shown that placing the dropout layer between encoders and decoders is sufficient to let the whole network be able to make Bayesian inference. In our work, we test this strategy on a regression network with residual blocks. Our result also shows that putting dropout layers between residual blocks is sufficient to make Bayesian inference.

When the robot is executing IBVS tasks, it may happen that the robot TCP and the hand position are in the space that the training data do not cover when the distance between hand and TCP is in the critical range. Using a deterministic NN could predict undesired repulsive position, which will lead the robot to be out of its reach range. We perform two experiments to demonstrate that Bayesian DNN can avoid the undesired repulsive pose. First, we create new datasets to evaluate the regression ResNet and the Bayesian DNN when the hand is in the unseen space, TCP is in the unseen space, and both are in the unseen space. The results show that when the robot TCP is in the unseen space, the RMSE of ResNet is higher than that in the seen space, and the PICP of Bayesian DNN are also lower. This means that the predicted repulsive position will have higher uncertainty when TCP is in the unseen space. When the hand is in the unseen space, the predicted repulsive positions are less affected. Second, we test on a real robot with 50 TCPs which are not in the unseen space. 22 repulsive poses based on regression ResNet predictions are out of the robot reach range. All repulsive poses based on Bayesian DNN predictions are within the reach range.

We further test the convergence speeds of Bayesian DNN and the opposite repulsive pose. We test with 20 TCPs that the Bayesian DNN converges significantly faster than the opposite repulsive pose. On average, the opposite repulsive pose needs 667 more iterations until the sse is lower than 0.005. By moving to the opposite repulsive pose, the robot will take a longer time to move to the desired pose. Using Bayesian DNN will decrease IBVS time compared to using opposite repulsive pose. It can increase the efficiency of the robotic task.

Although the Bayesian regression ResNet is trained for faster convergence of the IBVS in our case, it is not limited to the IBVS case. The Bayesian DNN approach can be adapted to other approaches. In our work, the Bayesian regression ResNet is trained with the ground truth which has the least image feature error. This criterion can be changed when a different approach is used. For instance, when using PBVS instead of IBVS, the ground truth can be the repulsive pose candidate with the least object pose error.

## 7 Conclusion and Future Work

In this work, we describe a system for collision avoidance when the robot is executing IBVS tasks. When a hand is detected under the critical distance to the robot TCP, the robot will avoid the collision by moving to a repulsive pose. The repulsive pose is determined based on a Bayesian DNN, that is, a Bayesian regression ResNet. The Bayesian DNN allows the IBVS to converge faster than the opposite repulsive pose. It can also prevent the robot from moving to undesired positions when the robot TCP is not in the space that the training data cover.

Currently, we use the YOLO object detector to obtain the bounding box of the hand and use Kalman filter to predict the movement of the bounding box. As soon as the predicted box is within the critical distance, we move the robot to a repulsive pose. If the user is using two hands near the robot, when one of the hands falls in the critical distance with regard to the robot TCP, the repulsive action will be triggered. One improvement could be formulating a plane and deciding if the repulsive action will be triggered by evaluating the distance between the TCP and the plane. However, one situation that the human is intentionally approaching the robot TCP to perform tasks might exist. One track of future work is to use a deep learning model to recognize the intended human action which is under the critical distance. Furthermore, DNNs are also used in visual servoing (Bateux et al., 2018); another track of future work can be jointly training a deep model for visual servoing with the ability for collision avoidance.

## Data Availability

The raw data supporting the conclusions of this article will be made available by the authors, without undue reservation.
